# Automated monitoring of urine output in hospitalized patients with indwelling urinary catheters: a clinical evaluation on the cardiology ward

**DOI:** 10.1007/s12471-026-02038-6

**Published:** 2026-04-08

**Authors:** Jos Perdeck, Gilmer A. Sussenbach, Elliot N. Bradshaw, Ingeborg K. Go, Reinoud E. Knops, Tom F. Brouwer

**Affiliations:** 1 Department of Cardiology, Amsterdam UMC, Amsterdam, The Netherlands; 2 Department of Medical Innovation and Development, Amsterdam UMC, Amsterdam, The Netherlands

**Keywords:** Urine output, Diuresis, Monitoring, Urinary catheters, Electronic health record, Heart failure, Recompensation

## Abstract

**Background:**

Urine output (UO) is a key clinical parameter for assessing hemodynamic status, yet manual UO measurements are labour-intensive, and documentation is often incomplete and error-prone. The Gravity of Flow study evaluated an in-house developed urine production gauge (UPG), designed as a reusable-cost system compatible with standard urine bags for automatic, continuous UO measurement and Electronic Health Record (EHR) integration.

**Methods:**

This single-centre observational study enrolled 25 cardiology patients (519 h) with indwelling urine catheters. To assess completeness and volume accuracy, UPG performance was compared against standard manual urine charting and a 24-hour container (gold standard).

**Results:**

The UPG achieved superior median completeness compared to manual charting (100% vs. 40%, *p* < 0.001). The device demonstrated high accuracy (median absolute percentage error 2.3%) and minimal bias (median −3 mL), meeting ±5% equivalence criteria (78.3% within bounds). In contrast, manual charting failed to meet equivalence. Performance of the UPG remained consistent across various UO flow rates.

**Conclusion:**

By providing superior, automated UO data (accuracy, completeness, reliability), the UPG has the potential to optimise fluid management, enable earlier intervention, and reduce nursing workload. Future research should validate these benefits across various clinical settings and over longer periods.

## What’s new?

- The Urine Production Gauge (UPG) introduces a highly reliable, accurate an automated alternative to manual urine output monitoring.
- In routine clinical practice, the UPG achieved near-complete data capture and eliminated measurement error, providing superior time-series data compared to manual charting.
- Real-time monitoring via EHR integration could support the early recognition of fluid imbalance and clinical deterioration, enhancing fluid management strategies in cardiology patients where volume control is vital.

## Introduction

Urine output is a vital parameter routinely monitored in hemodynamically unstable hospitalised patients. A reduction in hourly urine output (UO), presenting as oliguria or anuria, can indicate hemodynamic instability arising from various clinical conditions, such as bleeding, infection, or heart failure [1–3]. Although invasive catheterisation is avoided in stable patients to prevent complications like urinary tract infections, it remains a prerequisite for precise hourly monitoring in high-risk populations. Unlike other vital signs, including heart rate, blood pressure, or temperature, urine output cannot be measured instantaneously. Instead, it requires continuous, time-dependent monitoring to provide meaningful clinical information.

The current standard of care (SOC) relies on manual hourly urine production measurements, which are often incomplete due to its labour-intensive nature and prone to rounding errors, thereby compromising accuracy, patient safety and clinical decision-making [4]. Commercial automated urine measurement systems have been previously developed, but often require costly hardware or proprietary disposables, limiting adoption.

To address this gap, the Flowsure project at Amsterdam University Medical Centre (AUMC) is developing a reliable, cost-effective, and reusable urine production gauge (UPG). The investigational UPG uses standard urine collection bags and low-cost components to reduce overall costs and enable wider use. It automatically and continuously records urine production and transfers these data to the electronic health record (EHR), thereby improving clinical care and decision-making while reducing nursing workload. Because the UPG is compatible with standard urine collection bags and eliminates the need for a separate measurement chamber, the disposables are lighter and use less plastic, reducing both waste and cost.

The objective of this study was to evaluate the in-house developed UPG in a clinical cardiology care setting. Its performance was assessed by comparing automated UO measurements against the current SOC, the manual urinometer (MU).

## Methods

### Study design

This was a prospective, single-centre, observational study conducted on the cardiology wards of the Amsterdam University Medical Centre (AUMC). Patient enrolment took place between March 2024 and April 2025. The study was approved by the local ethics committee, and all participants provided written informed consent.

A convenience sample of 25 participants was selected. This small sample size and exploratory design were chosen specifically to perform feasibility testing of the UPG device and to obtain initial data estimates necessary for hypothesis generation and the power calculation of the future adequately sized randomised controlled trials. As the primary objective was to assess the technical accuracy of the UPG, which is attached non-invasively to the urine collection bag, long-term clinical outcomes, such as catheter-associated urinary complications, were not recorded.

### Study population

Eligible patients were ≥ 18 years of age, admitted to the cardiology wards with an indwelling urinary catheter for standard clinical monitoring prior to study enrolment, and able to provide informed consent. Exclusion criteria were chronic anuria, known urological disorders, catheter dysfunction, anticipated catheter removal during the observation period, or a urine production rate of < 30 mL/h.

### Urine production gauge

**Fig. 1:**
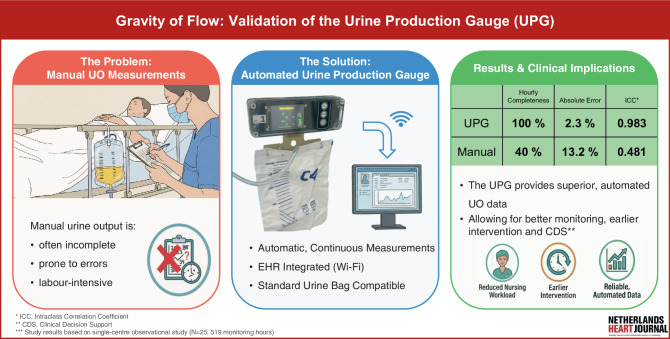
Infographic

The UPG is a reusable, in-house-developed device that measures urine production by tracking catheter bag weight over time using a load cell (Fig. 1). It features an integrated rechargeable battery and transmits measurements over Wi-Fi to the Electronic Health Record (EHR) through a custom HL7 server; the microcontroller includes a proprietary motion-artifact algorithm to filter noise and ensure stable, continuous measurements. As an in-house developed device, it currently has no CE mark and may be used only in approved clinical research.

The data transfer architecture was co-designed with an application architect and approved by internal governance. A data privacy impact assessment confirmed that privacy risks are adequately mitigated, and security measures ensure the protected transmission of the data.

Preclinical validation used a two-point calibration with infusion fluid (empty bag and 2 L), from which the calibration factor was derived. Linearity was confirmed with fixed weights, and functionality was tested by simulating urine production at varying infusion rates. As the load cell responds proportionally to any liquid, calibration with infusion fluid is appropriate. Given normal urinary specific gravity (1.003–1.030), applying a 1 g ≈ 1 ml conversion yields a clinically acceptable estimate, with a potential error margin of ~3% [5, 6].

### Measurement procedure

For each participant, urine output was recorded manually using a conventional manual urinometer (MU) and automatically with the UPG. Nurses documented the manually measured volume and timestamps on a worksheet, while the UPG logged data continuously. Observation periods lasted 8–24 h, during which all urine was drained into a 24-hour reference container serving as the gold standard for cumulative output.

At the start of each observation, collection bags were emptied, attached to the UPG, and nurses who had undergone group training were re-instructed on the study protocol at the start of each observation period and were instructed to brief their colleagues during subsequent shifts. A device manual was available on the ward.

Participation did not alter patient care. Patients could mobilise freely, and the catheter bag could be temporarily disconnected without affecting the study. The system detected weight absence during disconnection and, once reattached, computed the delta in urine volume, ensuring accurate output regardless of mobility.

### Outcome measures

The primary outcomes were (1) the completeness of hourly urine output registration, defined as the proportion of planned hourly measurements successfully captured by each method over a maximum of 24 h, and (2) the accuracy of cumulative urine output, determined by comparing volumes obtained with the MU and the UPG against the 24-hour reference standard.

### Statistical analysis

Descriptive statistics are reported as mean ± standard deviation (SD) or median with interquartile range (IQR), as appropriate. Completeness between UPG and manual documentation was compared using the Wilcoxon signed-rank test for paired samples. Completeness by shift was assessed with the same approach. Agreement between each method’s volume measurements and the gold standard was evaluated using Bland–Altman analysis, reporting bias, and 95% limits of agreement.

Accuracy was further assessed using absolute and signed percentage errors. Reliability was quantified using intraclass correlation coefficients (ICC) with 95% confidence intervals. Equivalence of UPG and manual methods against the gold standard was tested using two one-sided tests (TOST) within a ± 5% predefined margin. Subgroup analyses across strata of 24-hour urine volume (< 1000, 1000–2000, > 2000 mL/24 h) were limited to descriptive statistics, as group sizes were small and the study was not powered for inferential comparisons. Median values and interquartile ranges were reported for each subgroup. All analyses were performed in Python (version 3.13, PyCharm environment). Two-sided *p*-values < 0.05 were considered statistically significant.

## Results

### Study population

Twenty-five patients were enrolled, of whom two were excluded due to loss of their gold standard during the study period. The final analysis included 23 participants, contributing 519 monitored hours. The mean age was 72 ± 15.7 years, the mean weight was 72.8 ± 11.9 kg, and 52% were male. The primary admission diagnosis was decompensated heart failure in 16 patients (70%). At baseline, 18 patients (78%) received loop diuretics (17 furosemide, 1 bumetanide), and 6 (26%) were on mineralocorticoid receptor antagonists.

### Completeness of registration

The UPG achieved near-complete hourly data capture compared with manual charting. Median completeness was 100% (IQR 100–100%) for the UPG versus 40% (20.8–50.0%) for manual documentation, representing a median paired difference of 58.3 percentage points (Wilcoxon signed-rank *p* < 0.001). The advantage of the UPG was consistent across all nursing shifts (all *p* < 0.001) (Tab. 1).

**Table 1:** Completeness of hourly registration by shift

Category	Expected hours	UPG valid hours	UPG completeness (%)	Manual valid hours	Manual completeness (%)	*p*
Morning Shift	187.47	180	96.02	52	27.74	< 0.001
Afternoon Shift	156.50	156	99.68	82	52.40	< 0.001
Night Shift	176.17	176	99.91	50	28.38	< 0.001

### Accuracy

**Fig. 2:**
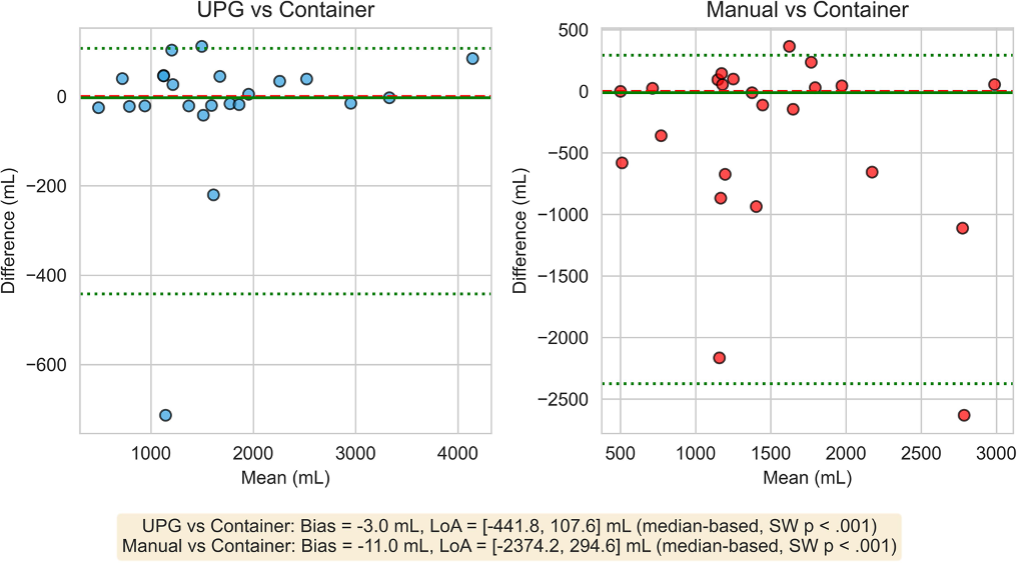
Bland-Altman Analysis of 24-Hour Urine Volume Measurement Methods

Compared with the gold standard, the UPG showed a minimal median bias of −3 mL (95% limits of agreement [LoA]: −442 to +108 mL) (Fig. 2). In contrast, manual charting demonstrated both a larger bias (−11 mL) and a substantially wider spread (LoA: −2374 to +295 mL). Notably, the UPG captured 105 non-zero measurements below 30 mL, compared to only 10 using manual charting.

The UPG demonstrated high precision, with a median absolute percentage error was 2.3% (IQR 1.4–4.6%), compared with 13.2% (4.2–40.9%) for manual charting. Signed percentage errors were close to zero for UPG (−0.1%, IQR −1.9 to 2.5%) but widely dispersed for manual charting (−0.8%, IQR −40.9 to 4.2%).

### Reliability

**Fig. 3:**
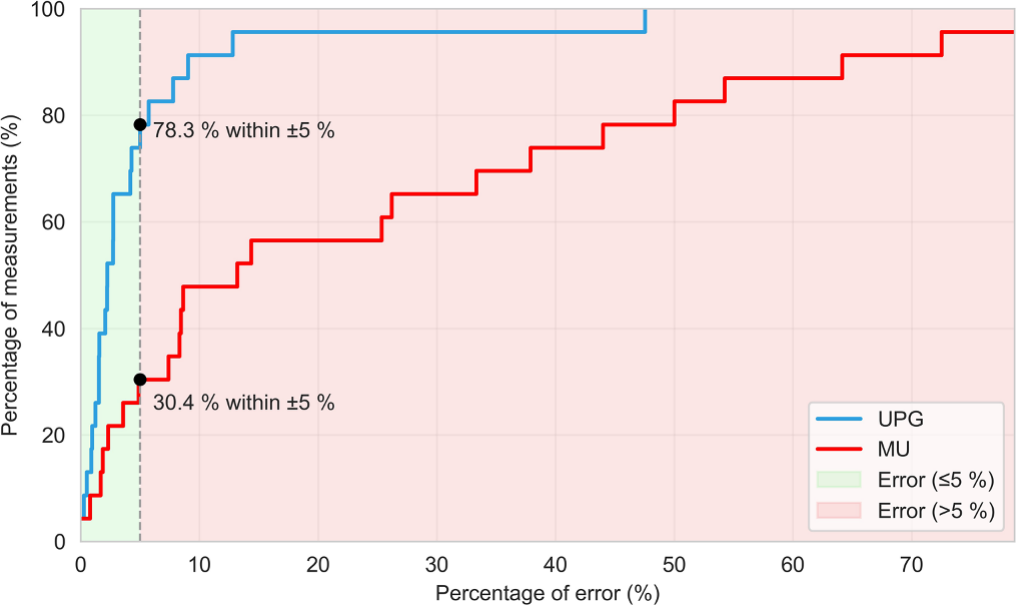
Accuracy Profile of 24-Hour Urine Volume Measurements

Agreement between UPG and the reference container was excellent (ICC 0.983, 95% CI 0.901–0.999), while manual charting showed poor agreement (ICC 0.481, 95% CI 0.163–0.821). Using a ± 5% equivalence margin, UPG met equivalence criteria (TOST *p* = 0.044), with 78.3% of measurements within bounds. Manual charting did not meet equivalence, with only 30.4% of measurements within bounds (Fig. 3).

### Subgroup analysis

Subgroup descriptives showed that UPG accuracy was consistent across all urine-output strata (< 1000, 1000–2000, > 2000 mL/24 h). In the low-output group (*n* = 4), the median absolute percentage error was 3.9%, compared with 2.7% in the medium group (*n* = 14) and 1.5% in the high group (*n* = 5), with absolute errors ranging from 24–44 mL. Manual charting showed markedly larger and more variable errors with absolute percentage errors of 20.7%, 8.5%, and 33.3% in the low-, medium-, and high-output groups, with corresponding absolute errors of 193 mL, 128 mL, and 1110 mL.

## Discussion

This study presents the first clinical evaluation of the investigational urine UPG, demonstrating significantly higher data completeness and accuracy than the SOC (median completeness 100% [IQR 100–100%] vs 40% [IQR 20.8–50%]). The UPG provided superior precision and was the only method to meet statistical equivalence within a ± 5% margin. Although a threshold of 30 mL/h was applied at inclusion, as device performance at very low rates was unverified, accuracy remained consistent across all volumes, including low-range output. One large deviation, attributable to incorrect taring, was intentionally retained and is visible as an outlier in Fig. 2 to reflect real-world use.

Manual measurement requires repeated, fixed-interval observations that often result in incomplete registration, especially during night and early-morning shifts, underscoring the value of automation [7]. Accurate UO data are central to clinical risk tools such as the MEWS and the SCAI cardiogenic-shock classification, where oliguria indicates impaired organ perfusion [8]. Automated registration could enhance early detection of deterioration through patient-specific alert thresholds. In conditions where urine output is a therapeutic target, such as acute decompensated heart failure, ESC guidelines call for a six-hourly review and therapy adjustment [9]; however, adherence is frequently limited by intermittent manual measurements. Automation supports guideline compliance, reduces nursing workload, and may enable more rapid, individualized therapy to alleviate symptoms, potentially shortening hospital stays.

Integrating continuous, high-resolution urine-output data into the EHR enables a new generation of fluid-management strategies. Automated protocols could track diuretic response in real time, balance decongestion against kidney-injury risk, detect anuria or oliguria immediately, and possibly reduce the need for frequent urinary sampling.

Although this study addressed short-term use, long-term implementation will depend on multi-day performance. The device used required no recalibration or maintenance, and EHR transmission was uninterrupted, supporting short-term stability but leaving durability in prolonged or higher-acuity settings unassessed. The device was designed to be cost-efficient, compatible with standard urine bags, and eliminate manual registration, thereby reducing nursing workload.

Currently, several automated urine output systems are CE-marked, such as Clarity RMS, Accuryn [10], and Sippy [11], offering some form of EHR integration. While each of these systems reduces manual workload and improves the UO registration, they all depend on custom disposables. In contrast, the system presented here operates with standard, off-the-shelf urine bags, while still providing EHR integration.

Limitations of this study include the small sample size (*n* = 23), single-centre design, and the fact that this represents initial clinical use. An early error in the sequence of zeroing during bag emptying caused substantial underreporting. Consequently, the user interface and instructional materials were refined, and nurse proficiency increased as the study progressed. Generalisability to longer monitoring and higher-acuity settings remains uncertain. As some authors and the institution hold shares in the developing company, analysis was performed by the non-financially involved first author, and an independent monitoring visit confirmed data integrity and protocol adherence to mitigate bias.

## Conclusion

The UPG offers a feasible alternative to manual urine output monitoring, combining high-fidelity data capture with low measurement error under routine clinical conditions. Its electronic health record integration and real-time data availability make it particularly valuable in cardiology, where precise fluid management is critical to the care of patients with acute decompensated heart failure, cardiogenic shock, or those in the perioperative phase of cardiac surgery. By supporting timely recognition of congestion and renal compromise, UPG monitoring has the potential to improve outcomes through earlier therapeutic intervention. The lessons learned from this first clinical evaluation have been used in the development of the next prototype. Future studies should also evaluate its impact on other patient populations, across different care settings, and over extended monitoring periods.
